# A Wideband High-Efficiency GaN MMIC Power Amplifier for Sub-6-GHz Applications

**DOI:** 10.3390/mi13050793

**Published:** 2022-05-20

**Authors:** Liulin Hu, Xuejie Liao, Fan Zhang, Haifeng Wu, Shenglin Ma, Qian Lin, Xiaohong Tang

**Affiliations:** 1 School of Electronic Science and Engineering, University of Electronic Science and Technology of China, Chengdu 611731, China; huliul@sina.com; 2 Chengdu Ganide Technology Company, Ltd., Chengdu 610220, China; liaoxuejie@foxmail.com (X.L.); zhangfan_uestc@163.com (F.Z.); abgott@126.com (H.W.); 3 Department of Mechanical and Electrical Engineering, Xiamen University, Xiamen 361005, China; mashenglin@xmu.edu.cn; 4 School of Physics and Electronic Information Engineering, Qinghai Minzu University, Xining 810007, China; linqian@tju.edu.cn

**Keywords:** MMIC, power amplifier, high efficiency, Sub-6-GHz, GaN/SiC HEMT

## Abstract

The monolithic microwave integrated circuit (MMIC) power amplifiers serve an essential and critical role in RF transmit/receive (T/R) modules of phased array radar systems, mobile communication systems and satellite systems. Over recent years, there has been an increasing requirement to develop wideband high-efficiency MMIC high power amplifiers (HPAs) to accommodate wideband operation and reduce power consumption. This paper presents a wideband high efficiency MMIC HPA for Sub-6-GHz applications using a 0.25-μm gate-length D-mode GaN/SiC high electron mobility transistor (HEMT) process. The amplifier consists of two stages with two HEMT cells for the driver stage and eight HEMT cells for the power stage. To obtain a flat gain while maintaining the wideband characteristic, a gain equalization technique is employed in the inter-stage matching circuit. Meanwhile, a low-loss output matching network is utilized to ensure high efficiency. The fabricated HPA occupies a compact chip area of 14.35 mm^2^ including testing pads. Over the frequency range of 2–6 GHz, measured results of this HPA show a saturated continuous wave (CW) output power of 44.4–45.2 dBm, a power added efficiency (PAE) of 35.8–51.3%, a small signal gain of 24–25.5 dB, and maximum input and output return losses of 14.5 and 10 dB, respectively.

## 1. Introduction

Gallium nitride (GaN), as one of the wide band-gap semiconductors, features a high electric breakdown field and high electron saturation velocity. Compared to the gallium arsenide (GaAs) and silicon (CMOS or LDMOS) PAs [1,2,3,4], GaN PAs exhibit higher output power, higher efficiency, wider bandwidth and better thermal characteristics. Therefore, GaN technology is a good candidate for realizing high performance HPAs [5,6,7,8,9,10,11,12].

Over recent years, to meet the demand of wideband operation and low power consumption for sub-6-GHz applications, wideband high-efficiency HPAs have been greatly desired and studied. Several fabricated wideband GaN HPAs have been reported in [13,14,15,16,17,18,19,20] to cover the frequency range of 2–6 GHz while maintaining watt-level output power. A 2–6 GHz two-stage high-efficiency GaN MMIC power amplifier based on gain compensation structure was implemented in [13] to deliver an output power of 35 dBm with a PAE larger than 45%. However, this amplifier suffers from poor input matching networks. A wideband two-stage MMIC HPA was presented in [14], with an output power of 40 dBm and a relatively low PAE of 25%. In [15], a 0.5–6.5 GHz non-uniform distributed GaN power amplifier with a small chip area was presented to obtain an output power higher than 30 dBm and a PAE of 20–38.1%. In [20], a 2.5–10.5 GHz GaN power amplifier with distributed and reactively-matched amplifier stages was implemented to achieve a saturated output power of 18–37 W and PAE of 19–40%. Nevertheless, the amplifier exhibits degraded return losses and relatively large chip size. To date, it is still a challenge to design a 2–6 GHz GaN HPA that simultaneously features better than 10 dB input/output return losses, 20 watt output power and more than 35% PAE.

In this work, a wideband high-efficiency HPA for sub-6-GHz applications using a 0.25-μm gate-length GaN/SiC HEMT process at a nominal power supply voltage of 28 V is developed and measured. Measurements of this chip show competitive performance in terms of better than 14.5 dB/10 dB input/output return losses, a 44.4–45.2 dBm (27.5–33 Watt) output power, and a 35.8–51.3% PAE in comparison to previously reported HPAs.

The rest of this paper is organized as follows. The utilized GaN HEMT technology and its transistor characteristics will be first described. This is followed by the design and analysis of the proposed HPA. The measured performances of the fabricated amplifier will be given and discussed before conclusion.

## 2. GaN HEMT Technology and Characteristics

**Figure 1:**
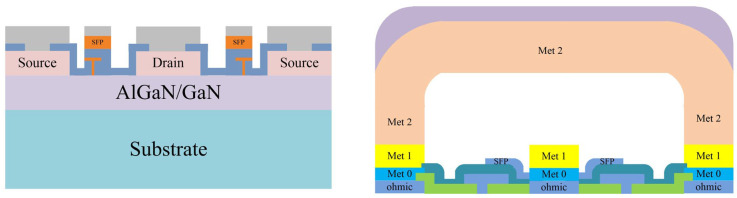
Schematic transistor cross-section of WIN Semiconductors’ 0.25μm GaN/SiC HEMT technology.

The two-stage PA is designed using a 0.25-μm gate-length D-mode GaN/SiC HEMT process on 100 μm SiC from WIN Semiconductors. The technology is suitable for high power applications from C-band through Ku-band. This process adopts a source-coupled field plate design to provide reliable operation breakdown voltage at high drain bias. Figure 1 demonstrates a representative transistor cross-section of the GaN HEMT process. The epitaxial layers were grown on top of the SiC wafer to constitute the HEMT and passive elements. The Au-metal layers consist of 0.6-μm MET0, 1.1-μm SFP, 1.1-μm MET1, and 4-μm MET2. The MET2 layer fulfills global interconnects to obtain low resistivity and high current handling capacity [21,22].

The HEMT of the GaN process features a cutoff frequency (*f*_T_) of 23 GHz and a maximum self-oscillation frequency (*f*_max_) of 65 GHz. Typical DC characteristics of the transistor are breakdown voltage exceeding 100 V at *I*_d_ = 1 mA/mm, and pinch-off voltage of −3.2 V, *I*_dmax_ = 1.05 A/mm, *G*_max_ = 340 mS/mm. The passive elements of the process include TaN thin film resistors with 50 Ω/square sheet resistivity, metal–insulator–metal (MIM) capacitors with capacitance density of 215 pF/mm^2^, round/square inductors, through-wafer vias for grounding, and air bridge crossover. The transistor and passive element models have been verified by measurements compared with simulations. Hence, the process design kit (PDK) models are accurate for our design in sub-6-GHz. The reliability information of the process can be referred to in [21].

**Figure 2:**
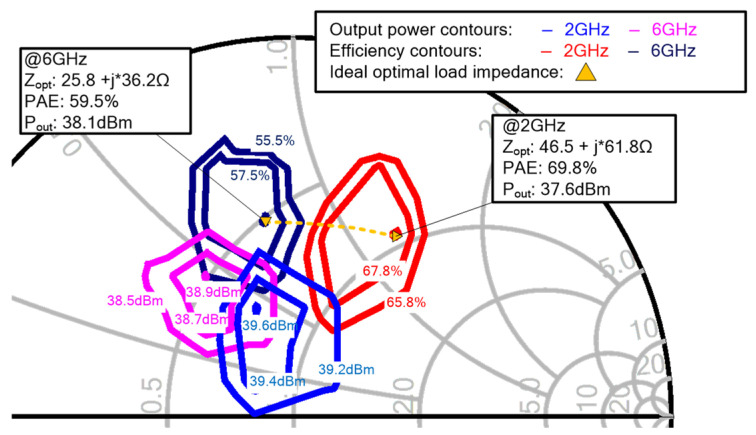
Load-pull contours for *P*_out_ and PAE from 2 GHz to 6 GHz and ideal optimal load impedance.

The presented amplifier consists of two stages with two HEMT cells (6 × 150-μm GaN HEMT) for the driver stage and eight HEMT cells (6 × 200-μm GaN HEMT) for the power stage. For each HEMT cell of the power stage, Figure 2 shows its load pull contours of *P*_out_, PAE and optimal load impedance at 2 and 6 GHz. The optimal load impedance of the transistor is chosen to approach the maximum PAE (>59.5%) while maintaining relatively large output power higher than 37.6 dBm at 6 GHz. It should be noted that the optimal load impedance is not constant across the frequency range of 2–6 GHz.

## 3. Power Amplifier Design

**Figure 3:**
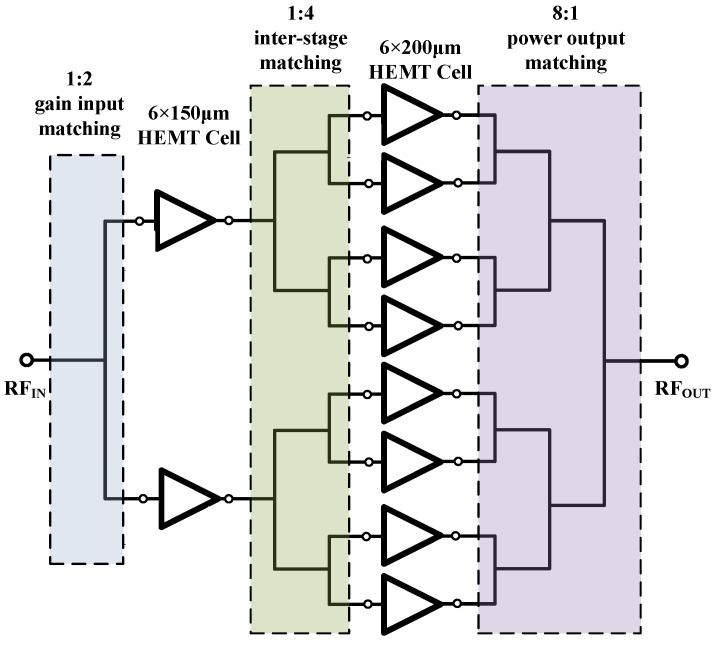
Functional block diagram of the presented sub-6-GHz MMIC HPA.

The motivation of this paper is to achieve a wideband high-efficiency MMIC PA with an output power of 44 dBm and a high PAE larger than 35% in the frequency range of 2–6 GHz. Figure 3 depicts the block diagram of the proposed two-stage PA. The total gate periphery is 11.4 mm from which the gate periphery ratios for the driver and power stages are equal to 3:16 to obtain sufficient driving power at the driver stage. The input matching and inter-stage matching circuits are designed to realize a good input match and a high gain with a good flatness, whilst the output matching circuit is selected to provide an optimal load match to obtain high efficiency and relatively large output power using the load/source pull simulation.

**Figure 4:**
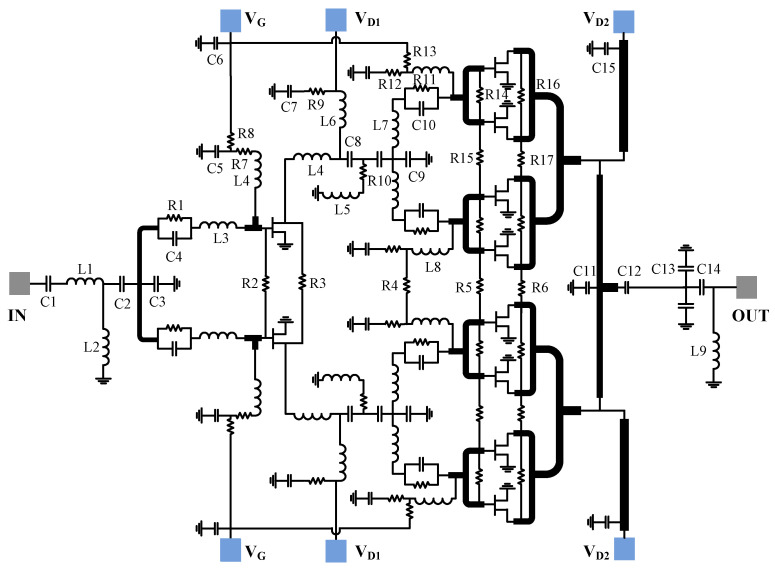
Detailed circuit diagram of the sub-6-GHz HPA.

**Figure 5:**
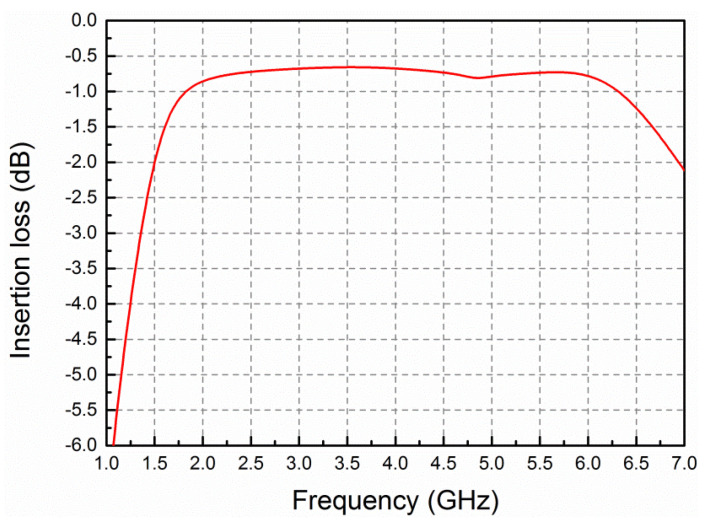
Simulated insertion loss of the output matching network against frequency.

Figure 4 shows the circuit schematic of the designed amplifier in detail. The input matching, inter-stage matching and output matching circuits are realized by both lumped elements and distributed circuits. The parameter values of the relevant lumped elements are listed in Table 1. The gate of each transistor is connected with a parallel-combined resistor and capacitor to guarantee unconditional stability over the entire frequency range. The resistors placed between adjacent parallel transistors are utilized to avoid odd-mode oscillation. To gain wideband characteristics and flatness, a gain equalization technique is employed in the inter-stage matching circuit, since a low-loss output matching circuit is critical for gaining high efficiency [23,24]. To minimize insertion loss, the output matching circuit consists of low-loss double-layer microstrip lines, a shunt inductor, as well as series and shunt high-quality factor MIM capacitors. It should be mentioned that a series inductor is not adopted in the output matching due to its low-quality factor. Figure 5 gives the simulated insertion loss of the output matching network. It is seen that the loss of the output matching circuit is 0.86–0.65 dB across the frequency range of 2–6 GHz, ensuring high efficiency and high output power of the amplifier. It is worth mentioning that extensive lumped elements employed in the input and inter-stage matching circuits are benefical in limiting chip size.

The amplifier adopts a class-AB bias point with drain voltage of 28 V and gate voltage of −2.4 V to improve efficiency. The driver and power stages share the same gate voltage pads while containing individual drain voltage pads. Additionally, inductors are employed to feed the DC power supply of the driver stage as well as the gate of the power stage, and microstrip lines are chosen to feed the drain of the power stage due to the heavy current.

## 4. Power Amplifier Measurement Results

**Figure 6:**
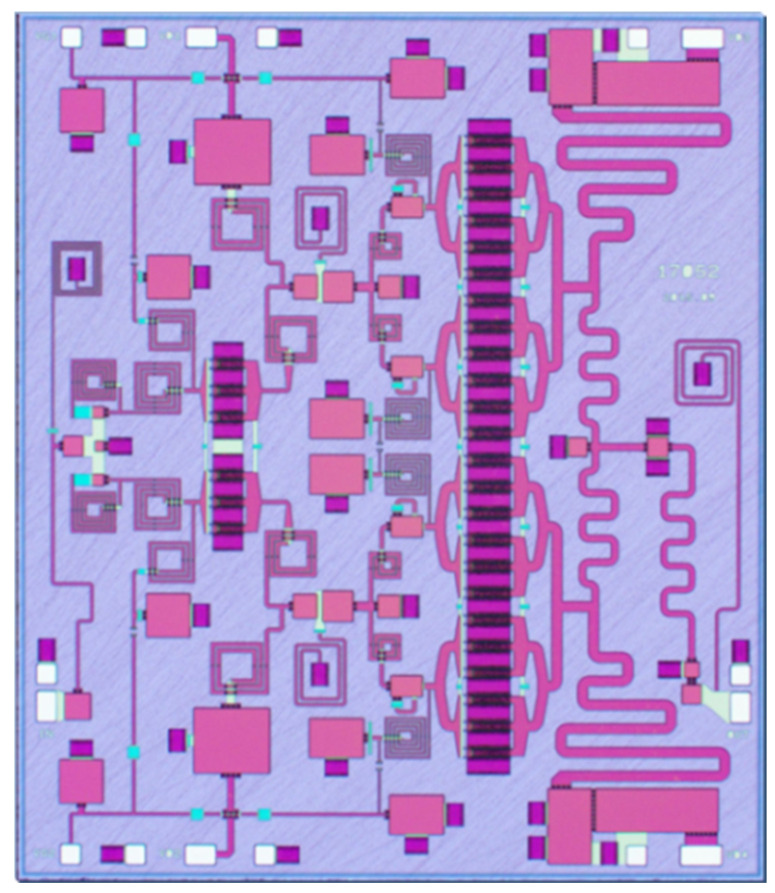
Microphotograph of the fabricated chip.

**Figure 7:**
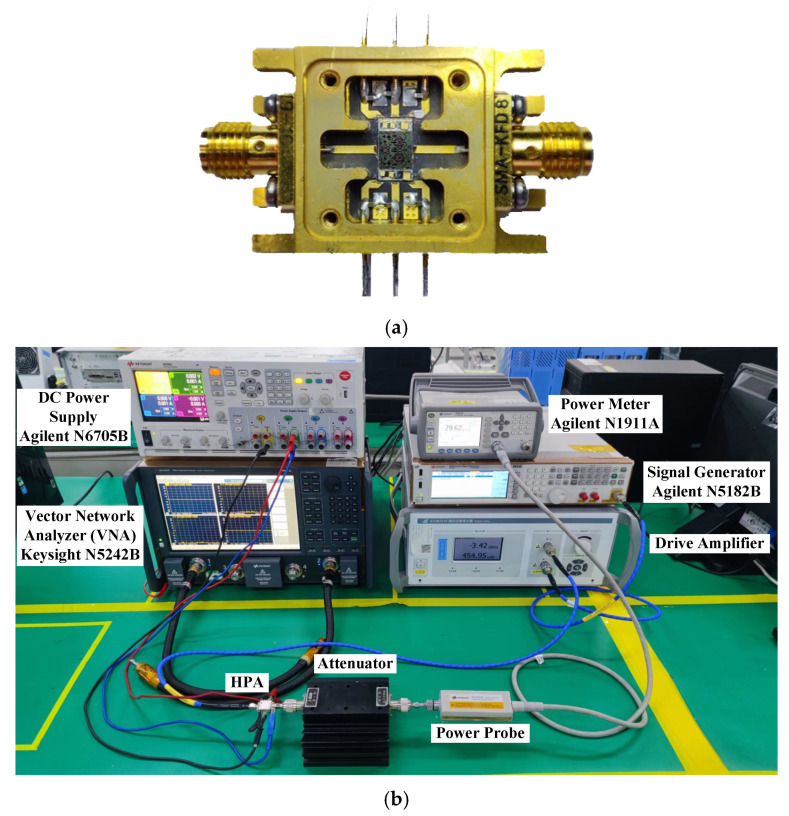
Photograph of the test fixture and small signal/large signal test environment of the HPA. (**a**) Test fixture; (**b**) test environment.

The two-stage HPA was implemented using a 0.25-μm gate-length D-mode GaN/SiC HEMT process. Figure 6 shows a microphotograph of the chip. Including testing pads, the chip occupies a die size of 3.5 mm × 4.1 mm with a SiC substrate thickness of 100 μm. To measure amplifier performance, the chip was mounted on a PCB board with bonding wires in a copper fixture as shown in Figure 7a. Each bonding wire is characterized with large inductance as a function of its length, and has small DC loss and capacitance. The amplifier was measured under CW conditions at the ambient temperature of 25 °C. The associated biased voltages are *V*_G_ = −2.4 V, *V*_D1_ = 28 V, *V*_D2_ = 28 V, and a 1.2-A quiescent DC current is supplied. The test environment of the amplifier is demonstrated in Figure 7b.

**Figure 8:**
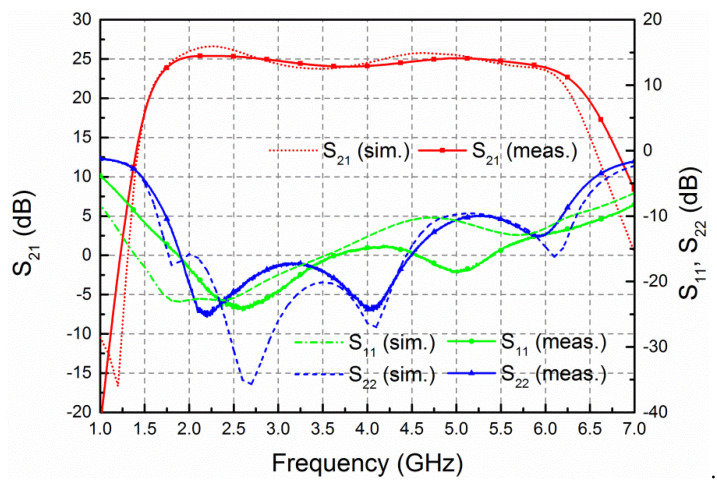
Simulated and measured S parameters.

The small-signal measurement of the HPA was completed via Keysight vector network analyzer (VNA) N5242 B (Keysight Technologies, Santa Rosa, CA, USA). Figure 8 demonstrates the measured S-parameters in comparison to the simulated ones. It is evident that the measurements and simulations are in good consistency. The amplifier achieves a small-signal gain of 24–25.5 dB with gain flatness less than ±0.75 dB across the frequency range of 2–6 GHz. The measured input and output return losses are better than 14.5 and 10 dB, respectively, achieving good input and output matching. 

**Figure 9:**
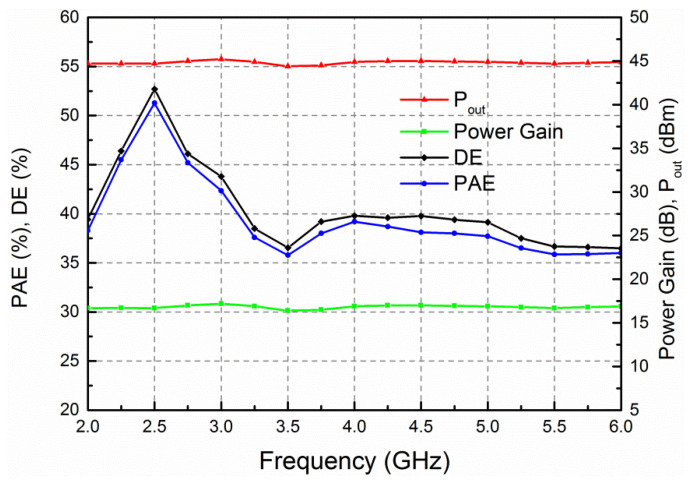
Measured saturated output power, DE, PAE and gain of the presented HPA against frequency with input fixed power of 28 dBm.

**Figure 10:**
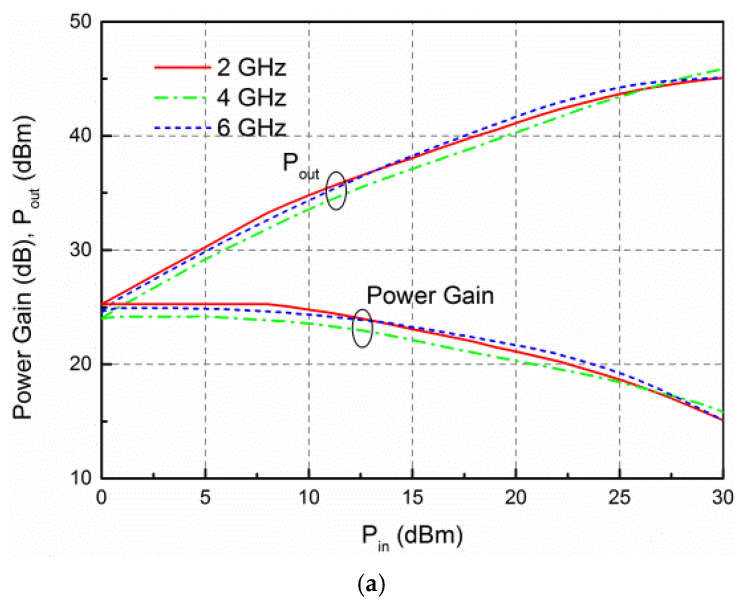

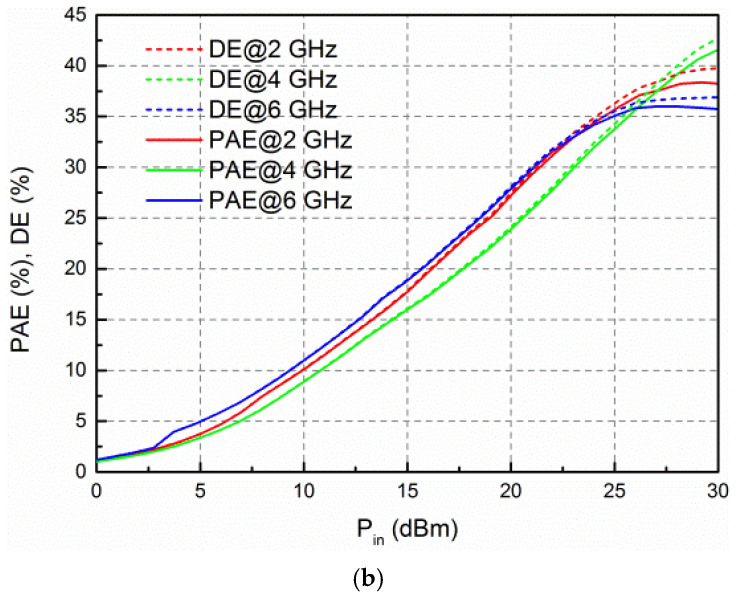
Large signal measured results of the presented HPA against input power at 2, 4 and 6 GHz. (**a**) Measured *P*_out_ and power gain against input power; (**b**) measured DE and PAE against input power.

The large-signal measurement was measured using an Agilent signal generator N5182 B (Keysight Technologies, Santa Rosa, CA, USA), drive amplifier, attenuator, and Agilent power meter N1911 A under driving CW signal. The measured saturated output power (*P*_out_), drain efficiency (DE), PAE, and gain against frequency are demonstrated in Figure 9. In this case, the input power (*P*_in_) is fixed as 28 dBm. The output power varies from 44.4 to 45.2 dBm, the DE and PAE are within 36.4–52.7% and 35.8–51.3%, respectively, and the power gain is between 16.4–17.2 dB over the band of interest. Figure 10 shows the measured *P*_out_, DE, PAE, and gain of the developed amplifier against input power at different frequencies. From 2 to 6 GHz, the *P*_out_ is 44.5–45 dBm with the associated DE greater than 36.7%, PAE higher than 35.7%, and power gain larger than 16.4 dB. 

Table 2 summarizes the performance comparison between this work and state-of the-art HPAs. It is obvious that the presented wideband amplifier exhibits competitive performance in terms of input/output return loss, output power and efficiency compared to previously reported wideband counterparts. 

## 5. Conclusions

This paper has reported the design and implementation of a wideband high-efficiency monolithic power amplifier suitable for sub-6-GHz applications utilizing a commercial 0.25-μm D-mode GaN HEMT process. The developed amplifier, with a compact chip area of 3.5 mm × 4.1 mm, demonstrates a delivered saturation output power of 44.4–45.2 dBm, a PAE higher than 35.8%, a small signal gain of 24–25.5 dB, and input and output return losses greater than 14.5 and 10 dB, respectively, over the entire 2–6 GHz bandwidth. It is believed that this outstanding MMIC power amplifier is promising and applicable for the T/R modules of sub-6-GHz systems due to its characteristics of wideband, good input/output match, high output power and high efficiency.

## Figures and Tables

**Table 1:** Parameter values of the lumped elements of the proposed HPA.

C1	C2	C3	C4	C5	C6	C7	C8	C9	C10	C11
4 pF	3.8 pF	1.5 pF	1.5 pF	2.3 pF	9 pF	35 pF	1.8 pF	0.2 pF	3 pF	1.1 pF
C12	C13	C14	C15	L1	L2	L3	L4	L5	L6	L7
2.2 pF	2 pF	2.2 pF	35 pF	1 nH	2.2 nH	1.3 nH	1 nH	3 nH	1.2 nH	1.5 nH
L8	L9	R1	R2	R3	R4	R5	R6	R7	R8	R9
2 nH	2.2 nH	35.6 Ω	38 Ω	19 Ω	544 Ω	38 Ω	18 Ω	76 Ω	544 Ω	50 Ω
R10	R11	R12	R13	R14	R15	R16	R17			
10 Ω	20 Ω	1.7 Ω	544 Ω	38 Ω	38 Ω	19 Ω	19 Ω			

**Table 2:** Comparison to previously published MMIC HPAs.

Ref.	Process	Stage	Freq. (GHz)	S_11_/S_22_ (dB)	*P*_out_ (dBm)	PAE (%)	DC Supply (V)	Die Area (mm^2^)
[2]	GaAs	1	1.5–10	<−9.5/<−10	30.7	33–44	7	4.62
[3]	GaAs	2	2–6.5	<−9.5/–	31–32	31.4–51.5	5	9.62
[4]	GaAs	2	0.5–6	<−13/<−15	29.5–31.1	22–29	12	4.8
[12]	GaN	1	4.6–5.5	<−7^+^/–	41.1–41.6	57.6–63.3	28	5.28
[13]	GaN	2	2–6	<−2/<−8	35	45	25	3.52
[14]	GaN	2	2–6	<−7/−	40	25	25	23.04
[15]	GaN	1	0.5–6.5	<−10/−7^+^	33.45	20–38.1	15	4
[16]	GaN	2	2–6	<−20/<−5	31.5	31	25	3.21
[17]	GaN	1	2–6	<−10/<−10	40.9–41.5	27–34	28	7.6
[18]	GaN	2	2.5–6	<−6/<−5	44–45.7	30.7–32.8	28	16.82
[19]	GaN	2	2–6	<−4/<−3	39	24–37	28	–
[20]	GaN	2	2.5–10.5	<−5^+^/<−4.5^+^	42.5–45.7	19–40	40	20
This work	GaN	2	2–6	<−14.5/<−10	44.4–45.2	35.8–51.3	28	14.35

Freq.: frequency; +: estimated value from figure.

## Data Availability

The presented data in this paper are available on request from the corresponding author.
